# Differential diagnosis of depression and Alzheimer’s disease with the Addenbrooke's Cognitive Examination-Revised (ACE-R)

**DOI:** 10.1186/s12883-015-0315-3

**Published:** 2015-04-17

**Authors:** Augustinas Rotomskis, Ramunė Margevičiūtė, Arūnas Germanavičius, Gintaras Kaubrys, Valmantas Budrys, Albinas Bagdonas

**Affiliations:** Vilnius University Faculty of Philosophy, Universiteto st. 9/1, Vilnius, Lithuania; School of Philosophy, Psychology and Language Sciences, The University of Edinburgh, Edinburgh, UK; Public Health Institute, Vilnius University, Vilnius, Lithuania; Clinic of Neurology and Neurosurgery, Faculty of Medicine, Vilnius University, Vilnius, Lithuania; Vilnius University Special Psychology Laboratory, Vilnius, Lithuania

**Keywords:** Alzheimer’s disease, Late-life onset depression with severe episode, Addenbrooke’s cognitive examination- revised, Differential diagnostics

## Abstract

**Background:**

One of the usual problems psychologists and clinicians face in clinical practice is differential diagnostics of Alzheimer’s disease and depression. It has been reported that the ACE and ACE-R could discriminate the cognitive dysfunctions due to depression from that due to dementia, although this is not uniform in all studies. The current study aimed to evaluate the utility of the ACE-R to differentiate late-life onset depression (with severe episode) from mild-moderate Alzheimer’s Disease (AD).

**Methods:**

This study received approval from the Lithuanian Bioethics Committee. All participants were older than 50 years (mean age = 66.52 (±8.76) years). The study sample consisted of 295 individuals: 117 with severe depression, 85 with mild-moderate Alzheimer’s disease (AD), and 94 age, gender and education matched participants of control group.

**Results:**

The ACE-R had high sensitivity (100%) and specificity (81%) at detecting cognitive impairments related to AD. Patients with late-life onset depression (ACE-R mean 76.82, SD = 7.36) performed worse than controls (ACE-R mean 85.08, SD = 7.2), but better than the AD group (ACE-R mean 54.74, SD = 12.19). Participants with late-life onset depression were differentiated by mild impairment in the ACE-R total score with mild memory (13.79, SD = 6.29) and greater deficits in letter fluency (3.65, SD = 1.21) than in semantic fluency (4.68, SD = 1.23). Participants with AD were differentiated by severely impaired performance on attention and orientation (11.80, SD = 2.93), memory (8.25, SD = 3.47) and language subtests (17.21, SD = 4.04), and moderately impaired performance on verbal fluency (6.07, SD = 2.74).

**Conclusions:**

ACE-R has diagnostic accuracy in detecting people with AD and can be used in differential diagnostics of late-life onset depression (severe episode) and AD. Diagnostic accuracy may be improved by analyzing the neuropsychological profiles and using lower cutoffs for different age groups.

## Background

The development of novel treatments for AD, aimed at ameliorating symptoms and modifying disease processes are likely to be the the most successful early in the disease, which increases the need for early diagnosis [1]. The early differential diagnosis between Alzheimer’s disease (AD) and late-life-onset depression remains a diagnostic challenge in neurology and psychiatry [2]. Patients presenting with combinations of cognitive, affective, and behavioral problems pose a clinical conundrum. In some cases, it is difficult to establish whether the cognitive impairment is secondary to an affective disorder, or to organic dementing process. A small proportion of depressed individuals present with significant cognitive dysfunction, formerly known as depressive pseudodementia, also termed “functional dementia”, “memory disorder in the context of depressive illness”, or “the dementia syndrome of depression” [3]. Accurate diagnosis is difficult in older adults for several reasons: patient and family members may give confusing and conflicting information; depression and dementia may be attributed to the normal effects of aging; dementia and depression often present as co-morbid conditions [3,4]. In clinical practice, differential diagnosis of severe depression from early dementia remains difficult, which leads to misdiagnosis of severe depression as early dementia.

The differential diagnosis of early stage AD and late life onset severe depression is especially difficult, because these diseases greatly overlap in cognitive impairments. Mild AD is characterized in the early stages by deficits in episodic memory [5], which are particularly clear on tasks requiring learning and retention of either verbal or nonverbal information [6,7]. However, patients with severe depression also tend to perform poorly on both verbal and nonverbal memory tests [8-10]. To complicate matters even further, difficult verbal episodic memory tasks such as recall tasks often fail to discriminate accurately severe depression patients from mild AD patients [11].

One important aspect of the clinical assessment of cognitive impairment in depression should be identifying cognitive screening instruments that differentiate the cognitive deficits most characteristic of depression from those that are most likely to reflect AD [12]. Unfortunately, there are few empirical data on screening measures that effectively discriminate between the cognitive presentations of AD and depression. In a study that illustrates the diagnostic problem, the Short Cognitive Evaluation Battery was found to demonstrate 94% sensitivity and 85% specificity for discriminating AD from non-demented and non-depressed controls, but there was only 63% sensitivity with 96% specificity discriminating AD from individuals with depression symptoms [13]. Similar issues of test insensitivity in discriminating depression are present in the Mini-Mental State Examination [14], which is widely used to estimate the severity of cognitive impairment, but which is less sensitive to milder cognitive impairment that might be expected in depression because of a low ceiling of difficulty, narrow range of cognitive abilities assessed, and differential sensitivity to age, education and ethnicity [15]. Based on current evidence, there are no screening measures that are sufficiently valid for distinguishing among depression and AD in a clinical setting, and this is even more of an issue when depression and cognitive impairment occur together. There is a need for multidimensional and easily accessible dementia screening tools that would accurately identify people suffering from AD and differentiate them from those suffering from depression.

The Addenbrooke‘s Cognitive Examination – Revised (ACE-R) is a brief cognitive dementia screening test battery recently adapted to Lithuanian population, which could be recommended as the most appropriate tool for dementia screening and possibly differential diagnosis from depression. It has been reported that the ACE-R could discriminate the cognitive dysfunctions due to depression from that due to dementia [16], although this is not uniform in all cultural backgrounds [17]. We argue that ACE-R can be used to differentiate depression from AD. For the ACE-R to be in differential diagnostics of depression and AD, culture specific cutoff scores have to be adjusted [18-20]. The Lithuanian language is different from English in the length of its words. Lithuanian words have more syllabeles than their English equivalents. This could explain the lower cut-off poins for the ACE-R in the Lithuanian-speaking population. In the validation study of version of the ACE-R in the Lithuanian-speaking population we identified a lower cuf-off score of 74 for the detection of dementia [21]. When the lower cut-off score of 74 was used, the sensitivity of the ACE-R to detect dementia was 91%. We argue that the Lithuanian version of ACE-R with a lower cut-off score of 74 for dementia could be used for differential diagnosis of AD and depression. Our study sought to investigate the ability of the ACE-R to accurately differentiate mild-moderate AD from severe depression.

## Methods

### Participants

We recruited the following participants: 85 participants with early mild-moderate AD, 117 participants with late-life onset depression (with severe episode), and 94 healthy controls. Consecutive referrals to the Neurology Department of the Vilnius University Hospital Santariskiu Clinics were screened for possible inclusion into the study. Outpatients with late-life onset depression (with severe episode) fulfilling the criteria for participation in our study were recruited from the Vilnius City Mental Health Centre. The inclusion criteria for all the groups are displayed in Table 1. Participants were excluded from the study, if they had a concurrent degenerative CNS disease (for example, Parkinson’s disease) or other primary nervous system diseases (for example, epilepsy), an acute stroke, primary psychiatric disorder (for example, schizophrenia), clinically significant kidney or liver disease, thyroid dysfunction or vitamin B12 deficiency. All participants were between 50 and 88 years old at the time of recruitment and were well matched for age, sex, and education. All participants had at least 4 years of education. Spouses or friends of the participating participants were recruited as healthy controls. All participants had sufficient knowledge of Lithuanian language to participate in the study. The majority of control participants were able to perform all of the tasks in the test. Participants who had visual problems were asked to wear glasses. None of the participants had severe hearing or other sensory impairments. The study and informed consent form was approved by the Lithuanian Bioethics Committee. Written informed consent for participation in the study was obtained from all participants.

**Table 1:** **Inclusion criteria for the participant recruitment**

	**AD group**	**Depression group**	**Control group**
The patient has probable AD diagnosed according to National Institute of Neurological and Communicative Disorders and Sroke and Alzheimer’s Disease and Related Disorders Association (NINCDS-ADRDA) criteria at the time of testing [34].	+		
The patient has had a CT or an MRI at the time of diagnosis establishment with results consistent with the diagnosis of probable AD (according to the mandatory standarts from the Lithuanian Health Ministry).	+		
The patient has a Mini Mental State Examination (MMSE) score at screening of at least 18, and not greater than 23 [35].	+		
Patients fulfilled International Classification of Mental and Behavioural Disorders Australian modification (ICD-10-AM) criteria [21] for severe depression episode at the time of testing (code: F32.20). All diagnoses of depression were established by experienced psychiatrist.		+	
The patient has a Mini Mental State Examination (MMSE) score at screening of at least 27 [35].			+

### Instrument

#### Addenbrooke’s Cognitive Examination-Revised (ACE-R)

The ACE-R is a brief, 15–20-min test battery originally designed to detect and classify different kinds of dementia, particularly AD and frontotemporal dementia, without the use of specialized test equipment [22]. The ACE-R takes between 12 and 20 min (average 16) to administer and score in a clinical setting. It contains 5 subtests, each one representing one cognitive domain: attention/orientation (18points), memory (26 points), fluency (14 points), language (26 points) and visuospatial (16 points). ACE-R maximum score is 100, composed by the addition of the all subtests.

### Statistical analysis

The statistical analysis was carried out with the SPSS for Windows package. The possible influence of demographic factors (age, gender and education) on the ACE-R scores was investigated. A general linear regression model was formed in order to test whether gender, age and education have an effect on the ACE-R test scores. To analyze the extent of utility of the ACE-R scores in prediction of the presence or absence of clinical diagnosis the binominal logistic regression analysis was used. We applied a receiver operating characteristic curve (ROC) analysis to examine the sensitivity and specificity of our measures. One-way ANOVAs and Mann–Whitney U-tests were carried out to compare the relevant group means of performance on the ACE-R test. The Chi square test was used to compare relevant group frequencies.

## Results

### Demographics

Demographic characteristics of the patient and control groups are summarized in Table 2. The groups were matched on age (one-way ANOVA, F[2, 293] = 0.154; p = 0.857), years of education (one-way ANOVA, F[2, 293] = 1.376; p = 0.254) and gender (χ^2^, p = 0.663).

**Table 2:** **Demographic characteristics of the patient and control groups**

	**Total**	**Mild-moderate AD**	**Late-life onset depression (with severe episode)**	**Controls**
Females in percent	64.5%	63.5%	67.5%	67.0%
Mean age in years (SD)	66.52 (±8.76)	66.33 (±7.92)	66.33 (±8.08)	66.93 (±10.26)
Years of education (SD)	11.48 (±3.33)	11.15 (±3.41)	11.36 (±3.59)	11.93 (±2.86)

*Note.* SD: Standard deviation.

To evaluate whether the demographic variables had an effect on performance on the ACE-R test scores, we formed general linear regression models for the patient and control groups. In AD group neither age (F = 1.288; Beta = −0.178; p = 0.110), nor gender (F = 7.588; Beta = −0.034; p = 0.759) nor education (F = 3.366; Beta = 0.149; p = 0.182) had an effect on ACE-R scores. In depression group both age had an effect (F = 12.111; Beta = −0.321; p < 0.001), while education (F = 0.339; Beta = −0.113, p = 0.211) and gender did not (F = 0.200; Beta = −0.061; p = 0.499). In control group both age (F = 2.174; Beta = −0.387; p < 0.001) and education (F = 2.869; Beta = 0.454; p < 0.001) had an effect, while gender did not (F = 0.101; Beta = 0.025; p = 0.769).

### ACE-R clinical utility

We carried out a logistic-regression analysis with two target variables: patients with AD group versus no-AD group (depression and healthy controls). The total ACE-R score correctly classified 93.9% of the cases.

**Figure 1:**
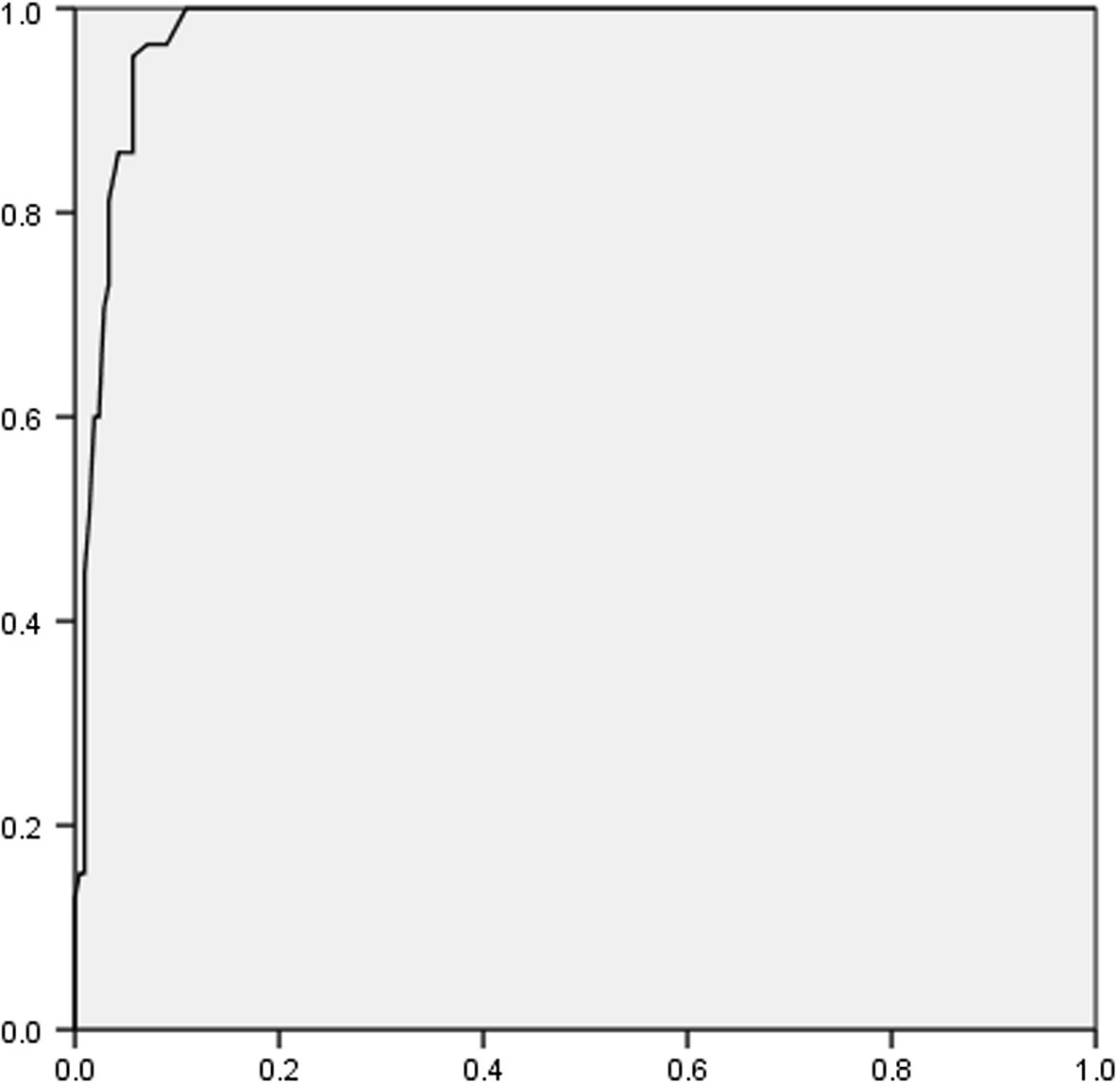
Receiver Operating Characteristics (ROC) of the Addenbrooke’s Cognitive Examination-Revised (ACE-R) as a Test for AD. Note. The blue line is the ROC curve, the black line is the diagonal line.

We carried out a ROC analysis with two target variables: patients with AD group versus no-AD group (depression and healthy controls). The trade-off between sensitivity (true positive rate) and 1–specificity (false positive rate) of the ACE-R in diagnosing AD in a patient population with and without a later confirmed AD dementia is shown in the ROC curve in Figure 1. The area under the ROC curve is 0.977, which suggests that the ACE-R has a high specificity for a large range of sensitivities. At 74, the previously recommended cut-off score [21] for clinical use in the detection of dementia, the ACE-R showed a sensitivity of 81%, and a specificity of 100% for AD in our study.

### ACE-R performance in participants with depression, AD and control groups

The mean scores on the ACE-R of the patient and control groups are shown in Table 3. A one-way ANOVA showed a significant between-group difference on ACE-R. To explore this further, we carried out post-hoc pairwise comparisons. The AD groups showed significant impairment relative to the control group (Bonferroni p < 0.001). The AD group showed significant impairment relative to depression group (Bonferroni; p < 0.001). The depression group showed significant impairment relative to control group (Bonferroni; p < 0.001). Participants with AD tend to fail the ACE-R (score below the recommended cut-off of 74 points) significantly more often than participants with severe depression or controls (chi square; p < 0.001). In addition, more participants with depression fail ACE-R than controls (chi square; p < 0.001). In the AD group 100% of participants fell below the cut-off score, in the depression group – 23.9%, in the control group – 8.5%.

**Table 3:** Addenbrooke’s Cognitive Examination - Revised (ACE-R) and its subtests means, and standard deviations in patient and control groups (in parenthesis SD)*

	**Mild-moderate AD**	**Late-life onset depression**	**Controls**	**F[df1,df2]**	**p value**
ACE-R total	54.74 (12.19)	76.82 (7.36)	85.08 (7.20)	271,7 [2, 293]	<0.001
Attention and orientation	11.80 (2.93)	17.00 (1.65)	17.65 (0.71)	307.98 [2, 293]	<0.001
Memory	8.25 (3.47)	13.79 (6.29)	18.52 (3.38)	93.05 [2, 293]	<0.001
Verbal fluency	6.07 (2.74)	8.33 (2.12)	10.56 (2.16)	83.19 [2, 293]	<0.001
Language	17.21 (4.04)	23.21 (2.73)	23.56 (2.17)	125.99 [2, 293]	<0.001
Visuospatial abilities	11.40 (2.70)	14.41 (1.64)	14.78 (1.69)	76.09 [2, 293]	<0.001

*Results of post-hoc analysis in detail are displayed in the text.

Because the depression group was likely to score statistically significantly lower than the control group, we ran a series of ANOVAs to test for differences between group performances in each of the ACE-R subtests. To explore this further, we carried out post-hoc pairwise comparisons. The AD groups showed significant impairment relative to the control group (Bonferroni p < 0.001). The impairment was severe, because the AD group scored in the range of more than three standard deviations below performance of controls. Also, the AD group showed significant impairment relative to depression group (Bonferroni; p < 0.001). The depression group showed no significant impairment relative to control group (Bonferroni; p = 0.067). The AD groups showed significant impairment relative to the control group (Bonferroni p < 0.001). The impairment was severe, because the AD group scored in the range of more than three standard deviations below performance of controls. Also, the AD group showed significant impairment relative to depression group (Bonferroni; p < 0.001). The depression group showed significant impairment relative to control group (Bonferroni; p < 0.001). The impairment was considered mild, because the depression group scored in the range of one to two standard deviations below the controls. The AD groups showed significant impairment relative to the control group (Bonferroni p < 0.001). The impairment was moderate, because the AD group scored in the range of two to three standard deviations below performance of controls. Also, the AD group showed significant impairment relative to depression group (Bonferroni; p < 0.001). The depression group showed significant impairment relative to control group (Bonferroni; p < 0.001). The impairment was considered mild, because the depression group scored in the range of one to two standard deviations below the controls. The AD groups showed significant impairment relative to the control group (Bonferroni p < 0.001). The impairment was mild, because the AD group scored in the range of two to three standard deviations below performance of controls. Also, the AD group showed significant impairment relative to depression group (Bonferroni; p < 0.001). The depression group showed significant impairment relative to control group (Bonferroni; p < 0.001). The impairment was considered mild, because the depression group scored in the range of one to two standard deviations below the controls. To explore this further, we carried out post-hoc pairwise comparisons. The AD groups showed significant impairment relative to the control group (Bonferroni p < 0.001). The impairment was severe, because the AD group scored in the range of more than three standard deviations below performance of controls. Also, the AD group showed significant impairment relative to depression group (Bonferroni; p < 0.001). The depression group showed no significant impairment relative to control group (Bonferroni; p = 1). To explore this further, we carried out post-hoc pairwise comparisons. The AD groups showed significant impairment relative to the control group (Bonferroni p < 0.001). The impairment was mild, because the AD group scored in the range of one to two standard deviations below performance of controls. Also, the AD group showed significant impairment relative to depression group (Bonferroni; p < 0.001). The depression group showed no significant impairment relative to control group (Bonferroni; p = 1).

Because we found mild impairments of orientation and attention, memory and verbal fluency subtests in the depression group, we compared the differences between the groups on memory and verbal fluency tasks. We used Mann–Whitney *U*-test to compare ACE-R performance of AD and depression groups. A series of Mann–Whitney U-tests revealed significant mean differences (Table 4) between the groups for all memory tasks. We ran a series of ANOVAs to test for differences between group performances in each of the verbal fluency subtest tasks. A one-way ANOVA showed a significant between-group difference on the letter fluency task (Table 5). The AD groups showed significant impairment relative to the control group (Bonferroni p < 0.001). Also, the depression group showed significant impairment relative to the control group (Bonferroni; p < 0.001). The AD group showed no significant impairment relative to the depression group (Bonferroni; p = 1). A one-way ANOVA showed a significant between-group difference on the category fluency task (Table 5). The AD groups showed significant impairment relative to the control group (Bonferroni p < 0.001). Also, the AD group showed significant impairment relative to the depression group (Bonferroni; p < 0.001). The depression group showed no significant impairment relative to the control group (Bonferroni; p < 0.001).

**Table 4:** **Means and standard deviations of memory subtest tasks in the AD and depression groups (in parenthesis SD)**

	**Mild-moderate AD**	**Late-life onset depression**	**p Value**	**Z-score**
Recall	0.8 (0.69)	1.65 (1.03)	<0.001	−5.894
Anterograde memory	3.53 (1.34)	4.40 (2.23)	<0.001	−3.612
Retrograde memory	1.87 (0.99)	2.56 (0.94)	<0.001	−4.684
Address recall	0.55 (0.89)	2.27 (2.28)	<0.001	−6.033
Address recognition	1.52 (1.18)	3.01 (1.64)	<0.001	−6.443

**Table 5:** Means and standard deviations of verbal fluency tasks in the subject groups (in parenthesis SD)*

	**Mild-moderate AD**	**Late-life onset depression**	**Controls**	**F[df1, df2]**	**p value**
Letter fluency	3.49 (1.52)	3.65 (1.21)	5.21 (1.25)	49,06 [2, 293]	<0.001
Category fluency	2.58 (1.45)	4.68 (1.23)	4.7 (1.3)	107,12 [2, 293]	<0.001

*Results of post-hoc analysis in detail are displayed in the text.

Because analysis of the neuropsychological profiles’ of depression and AD showed that these diseases have distinct neuropsychological impairments on the ACE-R, we invenstigated, how diagnostic accuracy may be improved by analyzing the neuropsychological profiles. A logistic-regression analysis using the domain scores of orientation, attention, category fluency, memory and language of the ACE-R again to predict membership in the target groups with and without a progressive-degenerative dementia indicated a satisfactorily high proportion, 97.8%, of the observed cases correctly predicted. A higher proportion of observed cases were correctly by using the identified domain scores.

## Discussion

The earlier studies established the Lithuanian version of Addenbrooke’s Cognitive Examination-Revised to be a sensitive and reliable tool to detect cognitive decline due to organic pathology [21]. We have extended this work by investigating, how ACE-R can be used in differential diagnostics in depression and AD.

The demographic differences had no effect on the study results, because the AD, depression and control groups did not differ significantly by age, gender or education. In our study age had a significant influence on ACE-R performance in depression and control groups (in line with previous findings by Margeviciute et al., [21]), which again emphasizes the need for age-specific ACE-R norms. Having the importance of age on overall performance in mind, it appears to be worthwhile to consider establishing different ACE-R cutoff points for the young-old and the old-old groups in Lithuanian-speaking population later on in the result analysis, as had been done in the Pigliautille and colleagues’ [23] adaptation of ACE-R. Future research in this field is needed to test this hypothesis.

In this study we have shown ACE-R to be a useful tool, which with high accuracy was able to detect participants with cognitive deficits due to AD and differentiate them from participants with late-life onset severe depression or healthy controls. The ACE-R displayed high clinical utility (high sensitivity and specificity). Despite this, participants with depression were likely to have a lower score in ACE-R than the control group. This pertained that participants with depression were statistically significantly more likely than the healthy controls to be identified as having dementia with ACE-R. This way we have replicated in our sample the problem of dementia-depression differentiation that is common in clinical practice. This led to a further analysis of the ACE-R subtests scores, how depression influenced the performance on ACE-R.

Further analysis of the neuropsychological profiles’ of depression and AD showed that these diseases have distinct neuropsychological impairments on the ACE-R. AD was characterized by severely impaired performance on attention and orientation, memory and language subtests, and moderately impaired performance on verbal fluency subtest. Meanwhile, mild impairment in the total ACE-R score, along with a low score on the memory and verbal fluency subtest tasks, characterized participants with depression. Memory and verbal fluency impairments found in depression are distinct from those found in AD. Memory deficits in AD group are different from those found in depression group. AD was characterized by more severe impairment of category fluency, while the depression was characterized by more severe selective impairment of letter fluency.

Considering the neuropsychological profile of the AD group on the ACE-R, it is consistent with the research on cognitive decline in early AD. Memory decline is the commonest complaint of participants and, more often, of their caregivers in AD. This is most commonly seen in the domain of anterograde episodic memory, that is the encoding storage, retention, and recall of new information about day-to-day personal experiences, which are accompanied by mild impairments in retrograde memory with a temporal gradient such that more distant memories are the most intact [24,25]. Consistent with previous research, we found accompanying deficits in attentional mechanisms, language and category fluency [25-27]. Considering visuospatial abilities in AD, visuoperceptual and visuospatial deficits are seldom clinically evident in the early stages of AD, with the notable exception of those participants who present with visual agnosia, the visual variant of AD [28]. This explains the occasionally found mild visuospatial deficits in AD group.

Considering the neuropsychological profile of depression, the results are mostly consistent with previous research. As expected memory impairment in AD seems to be more severe than in depression [29]. Memory tasks in the ACE-R could discriminate AD from depression. Although participants with late-life onset severe depression tended to score significantly lower than controls, they are likely to score statistically significantly higher than participants with AD. Moving to the verbal fluency findings, the depression group was characterized by letter fluency deficits and unimpaired semantic fluency, while in AD group category fluency deficits were significantly more severe than letter fluency deficits. Semantic memory impairments are detected in mild-moderate AD. On tests of verbal fluency, category fluency performance of patients with AD is more impaired than letter fluency, indicating difficulty accessing the semantic lexicon of word meanings [27]. In comparison to the extensive literature of letter- and category-based fluency in AD, relatively little attention has been paid to verbal fluency in affective disorders. Some researchers have found no significant difference between depressed and normal-comparison subjects [30], whereas others have reported impairment [31,32]. Studies consistent with our research have reported impaired letter but unimpaired semantic fluency in depressed elderly subjects [33], but more research is needed.

Although participants scoring below the cut-off score for dementia on ACE-R are likely to have AD not depression, we argue that these disorders should be differentiated by analyzing the neuropsychological profile of impairments. Mild impairment in the ACE-R total score with mild memory and greater deficits in letter fluency than in category fluency is indicative of depression not AD. Meanwhile, severe attention and orientation, memory and language deficits, greater deficits in semantic fluency than in letter fluency, and occasionally found mild impairments in visuospatial abilities are features attributable to AD.

## Conclusions

ACE-R has diagnostic accuracy in detecting people with AD and can be used in differential diagnostics of depression and AD. When interpreting the results it is important to compare the overall pattern of performance across all five subtests to verify the diagnosis, because depression and AD have distinct neuropsychological profiles. AD was characterized by severely impaired performance on attention and orientation, memory and language subtests, and moderately impaired performance on verbal fluency subtest. Meanwhile, mild impairment in the total ACE-R score, along with a low score on the memory and verbal fluency subtest tasks, characterized participants with depression. Memory and verbal fluency impairments found in depression are distinct from those found in AD. Memory deficits in depression are less severe than in AD, letter fluency is more impaired than category fluency, while in AD category fluency is more impaired than letter fluency. To sum up, ACE-R can be used in differential diagnostics of AD and depression.
